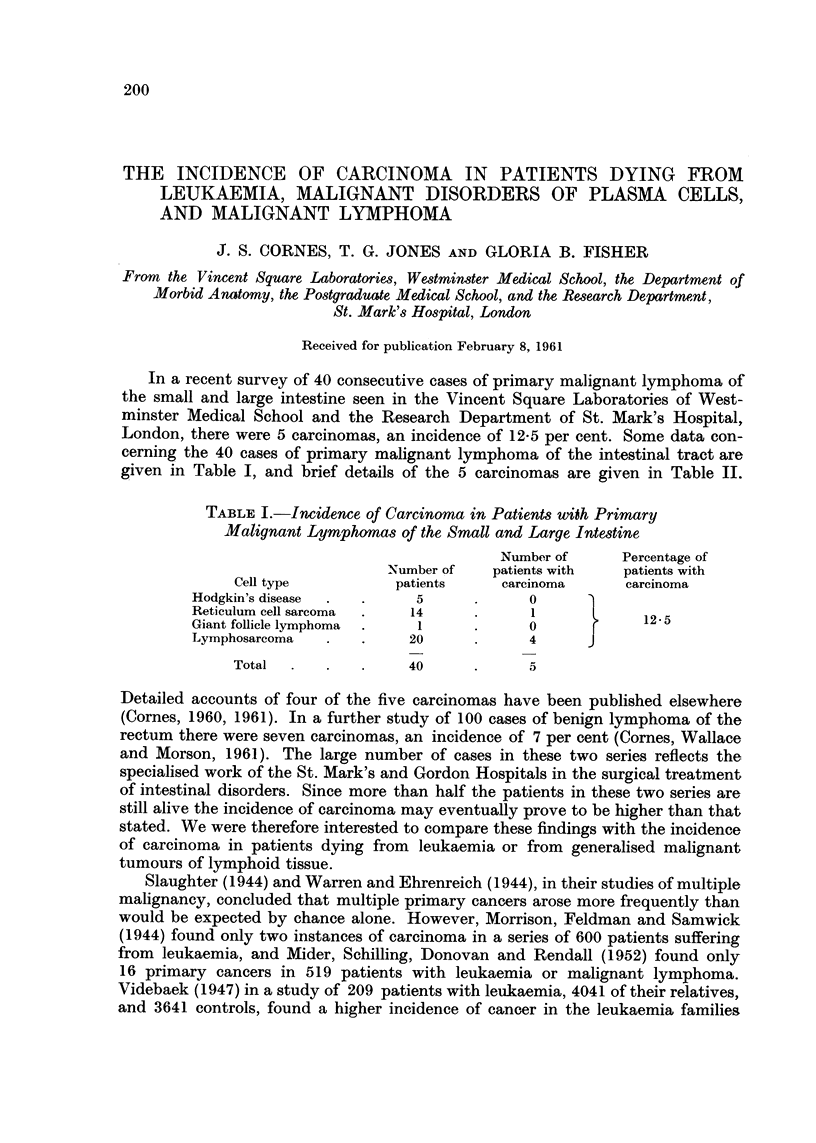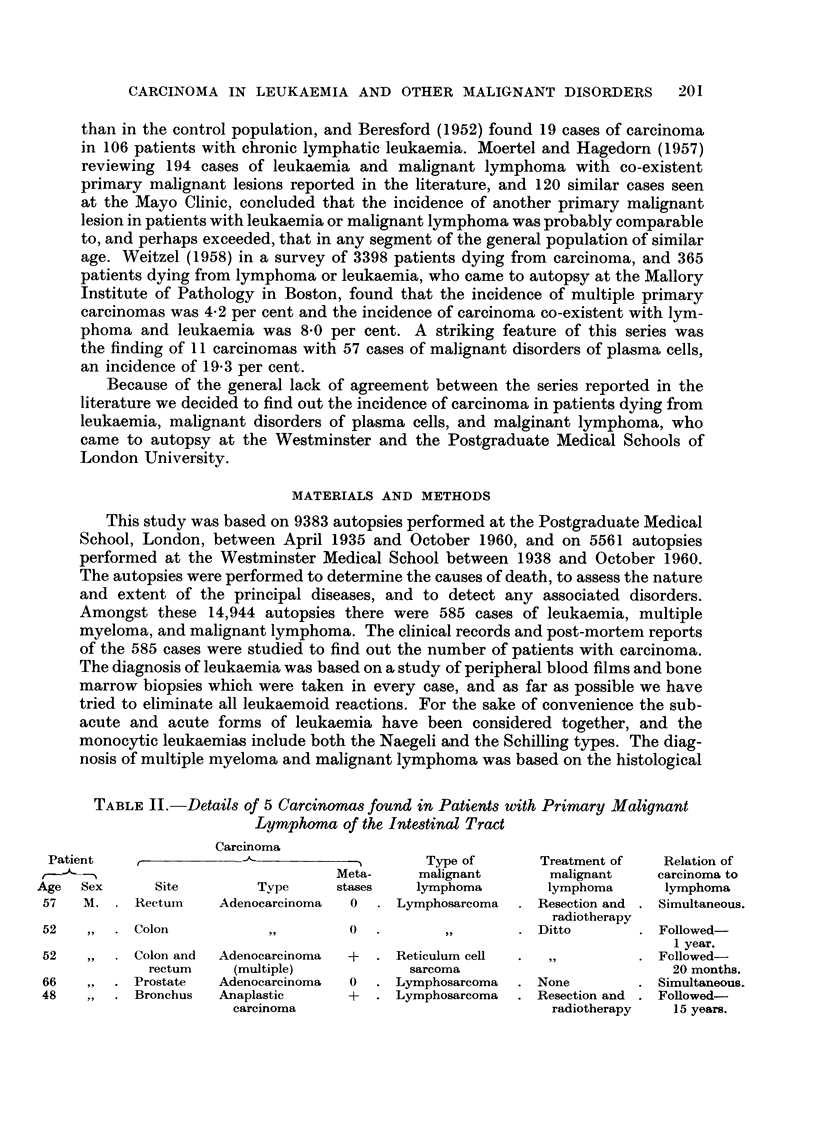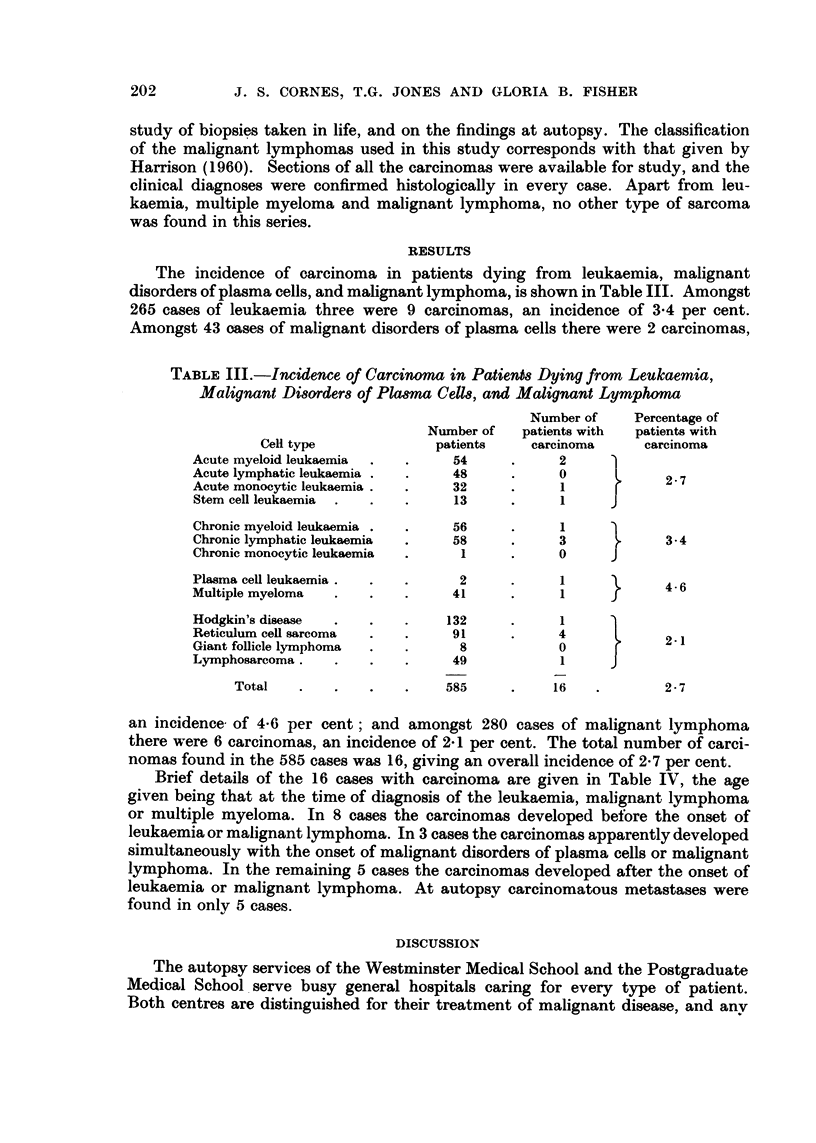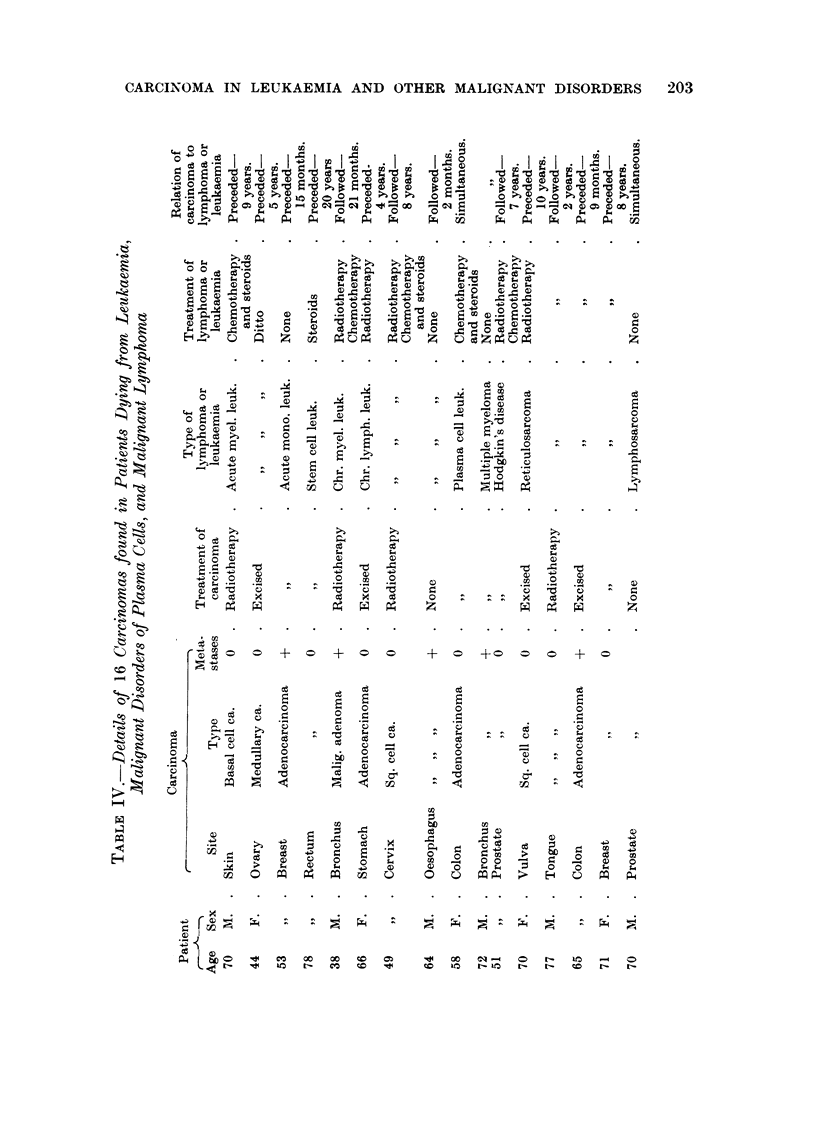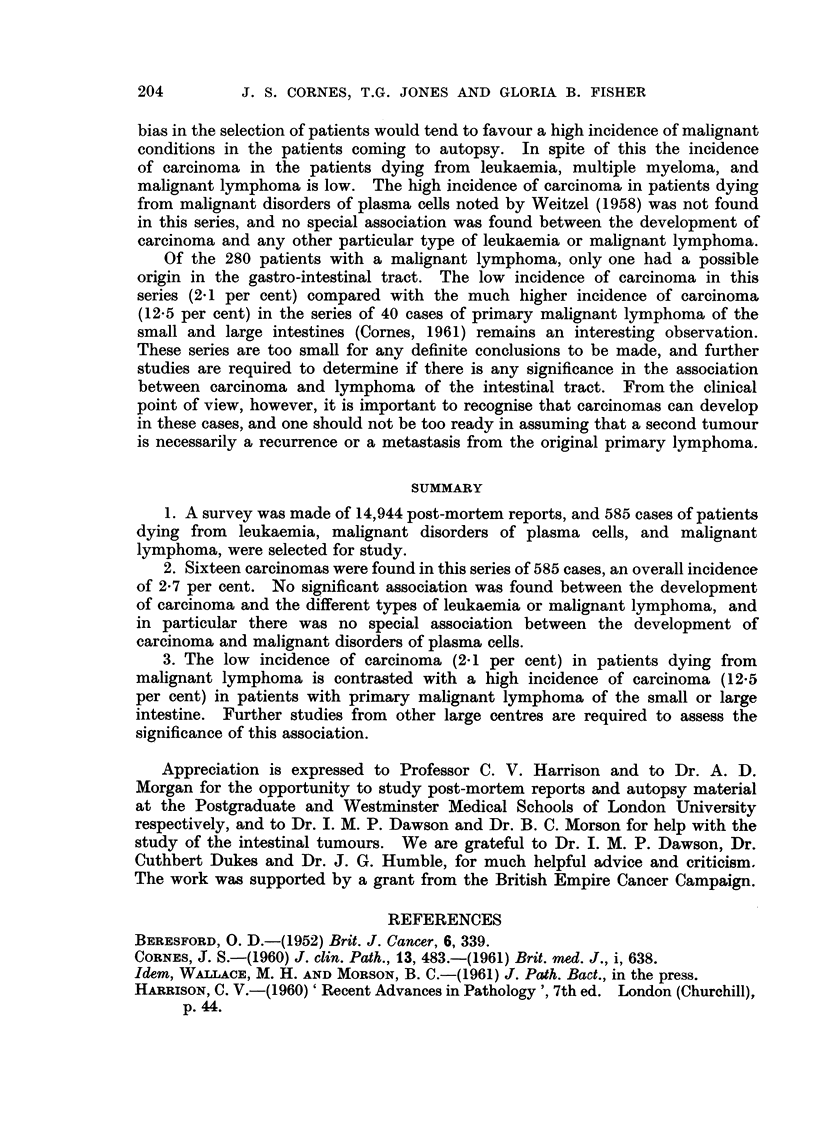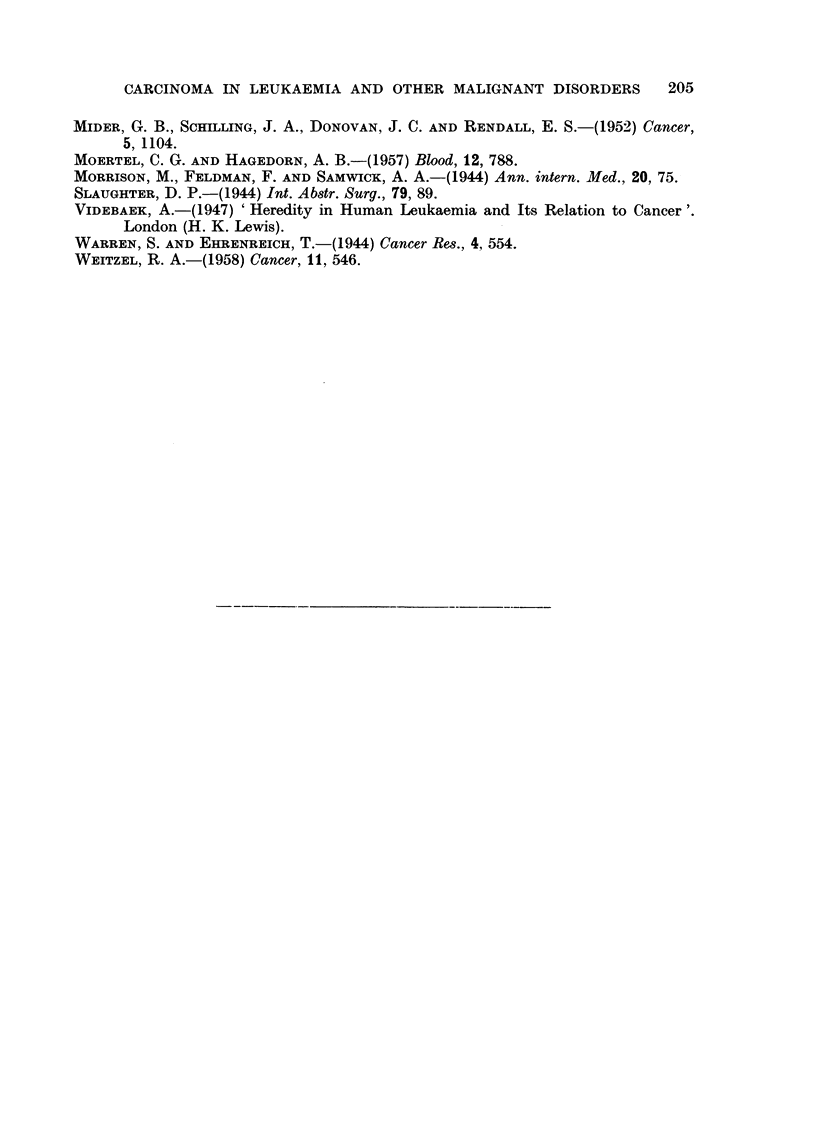# The Incidence of Carcinoma in Patients Dying from Leukaemia, Malignant Disorders of Plasma Cells, and Malignant Lymphoma

**DOI:** 10.1038/bjc.1961.24

**Published:** 1961-06

**Authors:** J. S. Cornes, T. G. Jones, Gloria B. Fisher


### THE INCIDENCE OF CARCINOMA IN PATIENTS DYING FROM LEUKAEMIA, MALIGNANT DISORDERS OF PLASMA CELLS, AND MALIGNANT LYMPHOMA

J. S. CORNES, T. G. JONES AND GLORIA B. FISHER

*From the Vincent Square Laboratories, Westminster Medical School, the Department of Morbid Anatomy, the Postgraduate Medical School, and the Research Department, St. Mark's Hospital, London*

Received for publication February 8, 1961

In a recent survey of 40 consecutive cases of primary malignant lymphoma of the small and large intestine seen in the Vincent Square Laboratories of Westminster Medical School and the Research Department of St. Mark's Hospital, London, there were 5 carcinomas, an incidence of 12·5 per cent. Some data concerning the 40 cases of primary malignant lymphoma of the intestinal tract are given in Table I, and brief details of the 5 carcinomas are given in Table II.

TABLE I.—*Incidence of Carcinoma in Patients with Primary Malignant Lymphomas of the Small and Large Intestine*

| Cell type | Number of patients | Number of patients with carcinoma | Percentage of patients with carcinoma |
|---|---|---|---|
| Hodgkin's disease | 5 | 0 | 12·5 |
| Reticulum cell sarcoma | 14 | 1 | |
| Giant follicle lymphoma | 1 | 0 | |
| Lymphosarcoma | 20 | 4 | |
| Total | 40 | 5 | |

Detailed accounts of four of the five carcinomas have been published elsewhere (Cornes, 1960, 1961). In a further study of 100 cases of benign lymphoma of the rectum there were seven carcinomas, an incidence of 7 per cent (Cornes, Wallace and Morson, 1961). The large number of cases in these two series reflects the specialised work of the St. Mark's and Gordon Hospitals in the surgical treatment of intestinal disorders. Since more than half the patients in these two series are still alive the incidence of carcinoma may eventually prove to be higher than that stated. We were therefore interested to compare these findings with the incidence of carcinoma in patients dying from leukaemia or from generalised malignant tumours of lymphoid tissue.

Slaughter (1944) and Warren and Ehrenreich (1944), in their studies of multiple malignancy, concluded that multiple primary cancers arose more frequently than would be expected by chance alone. However, Morrison, Feldman and Samwick (1944) found only two instances of carcinoma in a series of 600 patients suffering from leukaemia, and Mider, Schilling, Donovan and Rendall (1952) found only 16 primary cancers in 519 patients with leukaemia or malignant lymphoma. Videbaek (1947) in a study of 209 patients with leukaemia, 4041 of their relatives, and 3641 controls, found a higher incidence of cancer in the leukaemia families than in the control population, and Beresford (1952) found 19 cases of carcinoma in 106 patients with chronic lymphatic leukaemia. Moertel and Hagedorn (1957) reviewing 194 cases of leukaemia and malignant lymphoma with co-existent primary malignant lesions reported in the literature, and 120 similar cases seen at the Mayo Clinic, concluded that the incidence of another primary malignant lesion in patients with leukaemia or malignant lymphoma was probably comparable to, and perhaps exceeded, that in any segment of the general population of similar age. Weitzel (1958) in a survey of 3398 patients dying from carcinoma, and 365 patients dying from lymphoma or leukaemia, who came to autopsy at the Mallory Institute of Pathology in Boston, found that the incidence of multiple primary carcinomas was 4·2 per cent and the incidence of carcinoma co-existent with lymphoma and leukaemia was 8·0 per cent. A striking feature of this series was the finding of 11 carcinomas with 57 cases of malignant disorders of plasma cells, an incidence of 19·3 per cent.

Because of the general lack of agreement between the series reported in the literature we decided to find out the incidence of carcinoma in patients dying from leukaemia, malignant disorders of plasma cells, and malginant lymphoma, who came to autopsy at the Westminster and the Postgraduate Medical Schools of London University.

### MATERIALS AND METHODS

This study was based on 9383 autopsies performed at the Postgraduate Medical School, London, between April 1935 and October 1960, and on 5561 autopsies performed at the Westminster Medical School between 1938 and October 1960. The autopsies were performed to determine the causes of death, to assess the nature and extent of the principal diseases, and to detect any associated disorders. Amongst these 14,944 autopsies there were 585 cases of leukaemia, multiple myeloma, and malignant lymphoma. The clinical records and post-mortem reports of the 585 cases were studied to find out the number of patients with carcinoma. The diagnosis of leukaemia was based on a study of peripheral blood films and bone marrow biopsies which were taken in every case, and as far as possible we have tried to eliminate all leukaemoid reactions. For the sake of convenience the subacute and acute forms of leukaemia have been considered together, and the monocytic leukaemias include both the Naegeli and the Schilling types. The diagnosis of multiple myeloma and malignant lymphoma was based on the histological

TABLE II.—*Details of 5 Carcinomas found in Patients with Primary Malignant Lymphoma of the Intestinal Tract*

| Patient | | Carcinoma | | | Type of malignant lymphoma | Treatment of malignant lymphoma | Relation of carcinoma to lymphoma |
|---|---|---|---|---|---|---|---|
| Age | Sex | Site | Type | Metastases | | | |
| 57 | M. | Rectum | Adenocarcinoma | 0 | Lymphosarcoma | Resection and radiotherapy | Simultaneous. |
| 52 | ,, | Colon | ,, | 0 | ,, | Ditto | Followed—1 year. |
| 52 | ,, | Colon and rectum | Adenocarcinoma (multiple) | + | Reticulum cell sarcoma | ,, | Followed—20 months. |
| 66 | ,, | Prostate | Adenocarcinoma | 0 | Lymphosarcoma | None | Simultaneous. |
| 48 | ,, | Bronchus | Anaplastic carcinoma | + | Lymphosarcoma | Resection and radiotherapy | Followed—15 years. |

study of biopsies taken in life, and on the findings at autopsy. The classification of the malignant lymphomas used in this study corresponds with that given by Harrison (1960). Sections of all the carcinomas were available for study, and the clinical diagnoses were confirmed histologically in every case. Apart from leukaemia, multiple myeloma and malignant lymphoma, no other type of sarcoma was found in this series.

### RESULTS

The incidence of carcinoma in patients dying from leukaemia, malignant disorders of plasma cells, and malignant lymphoma, is shown in Table III. Amongst 265 cases of leukaemia three were 9 carcinomas, an incidence of 3·4 per cent. Amongst 43 cases of malignant disorders of plasma cells there were 2 carcinomas,

TABLE III.—*Incidence of Carcinoma in Patients Dying from Leukaemia, Malignant Disorders of Plasma Cells, and Malignant Lymphoma*

| Cell type | Number of patients | Number of patients with carcinoma | Percentage of patients with carcinoma |
|---|---|---|---|
| Acute myeloid leukaemia | 54 | 2 | 2·7 |
| Acute lymphatic leukaemia | 48 | 0 | |
| Acute monocytic leukaemia | 32 | 1 | |
| Stem cell leukaemia | 13 | 1 | |
| Chronic myeloid leukaemia | 56 | 1 | 3·4 |
| Chronic lymphatic leukaemia | 58 | 3 | |
| Chronic monocytic leukaemia | 1 | 0 | |
| Plasma cell leukaemia | 2 | 1 | 4·6 |
| Multiple myeloma | 41 | 1 | |
| Hodgkin's disease | 132 | 1 | 2·1 |
| Reticulum cell sarcoma | 91 | 4 | |
| Giant follicle lymphoma | 8 | 0 | |
| Lymphosarcoma | 49 | 1 | |
| Total | 585 | 16 | 2·7 |

an incidence of 4·6 per cent; and amongst 280 cases of malignant lymphoma there were 6 carcinomas, an incidence of 2·1 per cent. The total number of carcinomas found in the 585 cases was 16, giving an overall incidence of 2·7 per cent.

Brief details of the 16 cases with carcinoma are given in Table IV, the age given being that at the time of diagnosis of the leukaemia, malignant lymphoma or multiple myeloma. In 8 cases the carcinomas developed before the onset of leukaemia or malignant lymphoma. In 3 cases the carcinomas apparently developed simultaneously with the onset of malignant disorders of plasma cells or malignant lymphoma. In the remaining 5 cases the carcinomas developed after the onset of leukaemia or malignant lymphoma. At autopsy carcinomatous metastases were found in only 5 cases.

### DISCUSSION

The autopsy services of the Westminster Medical School and the Postgraduate Medical School serve busy general hospitals caring for every type of patient. Both centres are distinguished for their treatment of malignant disease, and any

TABLE IV.—*Details of 16 Carcinomas found in Patients Dying from Leukaemia, Malignant Disorders of Plasma Cells, and Malignant Lymphoma*

| Patient | | Carcinoma | | | | | | |
|---|---|---|---|---|---|---|---|---|
| Age | Sex | Site | Type | Metastases | Treatment of carcinoma | Type of lymphoma or leukaemia | Treatment of lymphoma or leukaemia | Relation of carcinoma to lymphoma or leukaemia |
| 70 | M. | Skin | Basal cell ca. | 0 | Radiotherapy | Acute myel. leuk. | Chemotherapy and steroids | Preceded—9 years. |
| 44 | F. | Ovary | Medullary ca. | 0 | Excised | ,, ,, ,, | Ditto | Preceded—5 years. |
| 53 | ,, | Breast | Adenocarcinoma | + | ,, | Acute mono. leuk. | None | Preceded—15 months. |
| 78 | ,, | Rectum | ,, | 0 | ,, | Stem cell leuk. | Steroids | Preceded—20 years |
| 38 | M. | Bronchus | Malig. adenoma | + | Radiotherapy | Chr. myel. leuk. | Radiotherapy Chemotherapy | Followed—21 months. |
| 66 | F. | Stomach | Adenocarcinoma | 0 | Excised | Chr. lymph. leuk. | Radiotherapy | Preceded-4 years. |
| 49 | ,, | Cervix | Sq. cell ca. | 0 | Radiotherapy | ,, ,, ,, | Radiotherapy Chemotherapy and steroids | Followed—8 years. |
| 64 | M. | Oesophagus | ,, ,, ,, | + | None | ,, ,, ,, | None | Followed—2 months. |
| 58 | F. | Colon | Adenocarcinoma | 0 | ,, | Plasma cell leuk. | Chemotherapy and steroids | Simultaneous. |
| 72 | M. | Bronchus | ,, | + | ,, | Multiple myeloma | None | ,, |
| 51 | ,, | Prostate | ,, | 0 | ,, | Hodgkin's disease | Radiotherapy Chemotherapy | Followed—7 years. |
| 70 | F. | Vulva | Sq. cell ca. | 0 | Excised | Reticulosarcoma | Radiotherapy | Preceded—10 years. |
| 77 | M. | Tongue | ,, ,, ,, | 0 | Radiotherapy | ,, | ,, | Followed—2 years. |
| 65 | ,, | Colon | Adenocarcinoma | + | Excised | ,, | ,, | Preceded—9 months. |
| 71 | F. | Breast | ,, | 0 | ,, | ,, | ,, | Preceded—8 years. |
| 70 | M. | Prostate | ,, | | None | Lymphosarcoma | None | Simultaneous. |

bias in the selection of patients would tend to favour a high incidence of malignant conditions in the patients coming to autopsy. In spite of this the incidence of carcinoma in the patients dying from leukaemia, multiple myeloma, and malignant lymphoma is low. The high incidence of carcinoma in patients dying from malignant disorders of plasma cells noted by Weitzel (1958) was not found in this series, and no special association was found between the development of carcinoma and any other particular type of leukaemia or malignant lymphoma.

Of the 280 patients with a malignant lymphoma, only one had a possible origin in the gastro-intestinal tract. The low incidence of carcinoma in this series (2·1 per cent) compared with the much higher incidence of carcinoma (12·5 per cent) in the series of 40 cases of primary malignant lymphoma of the small and large intestines (Cornes, 1961) remains an interesting observation. These series are too small for any definite conclusions to be made, and further studies are required to determine if there is any significance in the association between carcinoma and lymphoma of the intestinal tract. From the clinical point of view, however, it is important to recognise that carcinomas can develop in these cases, and one should not be too ready in assuming that a second tumour is necessarily a recurrence or a metastasis from the original primary lymphoma.

### SUMMARY

1. A survey was made of 14,944 post-mortem reports, and 585 cases of patients dying from leukaemia, malignant disorders of plasma cells, and malignant lymphoma, were selected for study.

2. Sixteen carcinomas were found in this series of 585 cases, an overall incidence of 2·7 per cent. No significant association was found between the development of carcinoma and the different types of leukaemia or malignant lymphoma, and in particular there was no special association between the development of carcinoma and malignant disorders of plasma cells.

3. The low incidence of carcinoma (2·1 per cent) in patients dying from malignant lymphoma is contrasted with a high incidence of carcinoma (12·5 per cent) in patients with primary malignant lymphoma of the small or large intestine. Further studies from other large centres are required to assess the significance of this association.

Appreciation is expressed to Professor C. V. Harrison and to Dr. A. D. Morgan for the opportunity to study post-mortem reports and autopsy material at the Postgraduate and Westminster Medical Schools of London University respectively, and to Dr. I. M. P. Dawson and Dr. B. C. Morson for help with the study of the intestinal tumours. We are grateful to Dr. I. M. P. Dawson, Dr. Cuthbert Dukes and Dr. J. G. Humble, for much helpful advice and criticism. The work was supported by a grant from the British Empire Cancer Campaign.

### REFERENCES

BERESFORD, O. D.—(1952) *Brit. J. Cancer*, **6**, 339.
CORNES, J. S.—(1960) *J. clin. Path.*, **13**, 483.—(1961) *Brit. med. J.*, i, 638.
*Idem*, WALLACE, M. H. AND MORSON, B. C.—(1961) *J. Path. Bact.*, in the press.
HARRISON, C. V.—(1960) ' Recent Advances in Pathology ', 7th ed. London (Churchill), p. 44.

MIDER, G. B., SCHILLING, J. A., DONOVAN, J. C. AND RENDALL, E. S.—(1952) *Cancer*, **5**, 1104.
MOERTEL, C. G. AND HAGEDORN, A. B.—(1957) *Blood*, **12**, 788.
MORRISON, M., FELDMAN, F. AND SAMWICK, A. A.—(1944) *Ann. intern. Med.*, **20**, 75.
SLAUGHTER, D. P.—(1944) *Int. Abstr. Surg.*, **79**, 89.
VIDEBAEK, A.—(1947) 'Heredity in Human Leukaemia and Its Relation to Cancer'. London (H. K. Lewis).
WARREN, S. AND EHRENREICH, T.—(1944) *Cancer Res.*, **4**, 554.
WEITZEL, R. A.—(1958) *Cancer*, **11**, 546.